# mTOR hyperactivity and *RICTOR* amplification as targets for personalized treatments in malignancies

**DOI:** 10.3389/pore.2024.1611643

**Published:** 2024-03-07

**Authors:** Dániel Sztankovics, Dorottya Moldvai, Gábor Petővári, Titanilla Dankó, Fatime Szalai, Risa Miyaura, Viktória Varga, Noémi Nagy, Gergő Papp, Judit Pápay, Ildikó Krencz, Anna Sebestyén

**Affiliations:** Department of Pathology and Experimental Cancer Research, Semmelweis University, Budapest, Hungary

**Keywords:** mTOR, mTORC2 hyperactivity, *RICTOR* amplification, Rictor overexpression, malignancies

## Abstract

The increasing knowledge of molecular alterations in malignancies, including mutations and regulatory failures in the mTOR (mechanistic target of rapamycin) signaling pathway, highlights the importance of mTOR hyperactivity as a validated target in common and rare malignancies. This review summarises recent findings on the characterization and prognostic role of mTOR kinase complexes (mTORC1 and mTORC2) activity regarding differences in their function, structure, regulatory mechanisms, and inhibitor sensitivity. We have recently identified new tumor types with *RICTOR* (rapamycin-insensitive companion of mTOR) amplification and associated mTORC2 hyperactivity as useful potential targets for developing targeted therapies in lung cancer and other newly described malignancies. The activity of mTOR complexes is recommended to be assessed and considered in cancers before mTOR inhibitor therapy, as current first-generation mTOR inhibitors (rapamycin and analogs) can be ineffective in the presence of mTORC2 hyperactivity. We have introduced and proposed a marker panel to determine tissue characteristics of mTOR activity in biopsy specimens, patient materials, and cell lines. Ongoing phase trials of new inhibitors and combination therapies are promising in advanced-stage patients selected by genetic alterations, molecular markers, and/or protein expression changes in the mTOR signaling pathway. Hopefully, the summarized results, our findings, and the suggested characterization of mTOR activity will support therapeutic decisions.

## Regulatory role of mTOR and its hyperactivity in cancer

**FIGURE 1:**
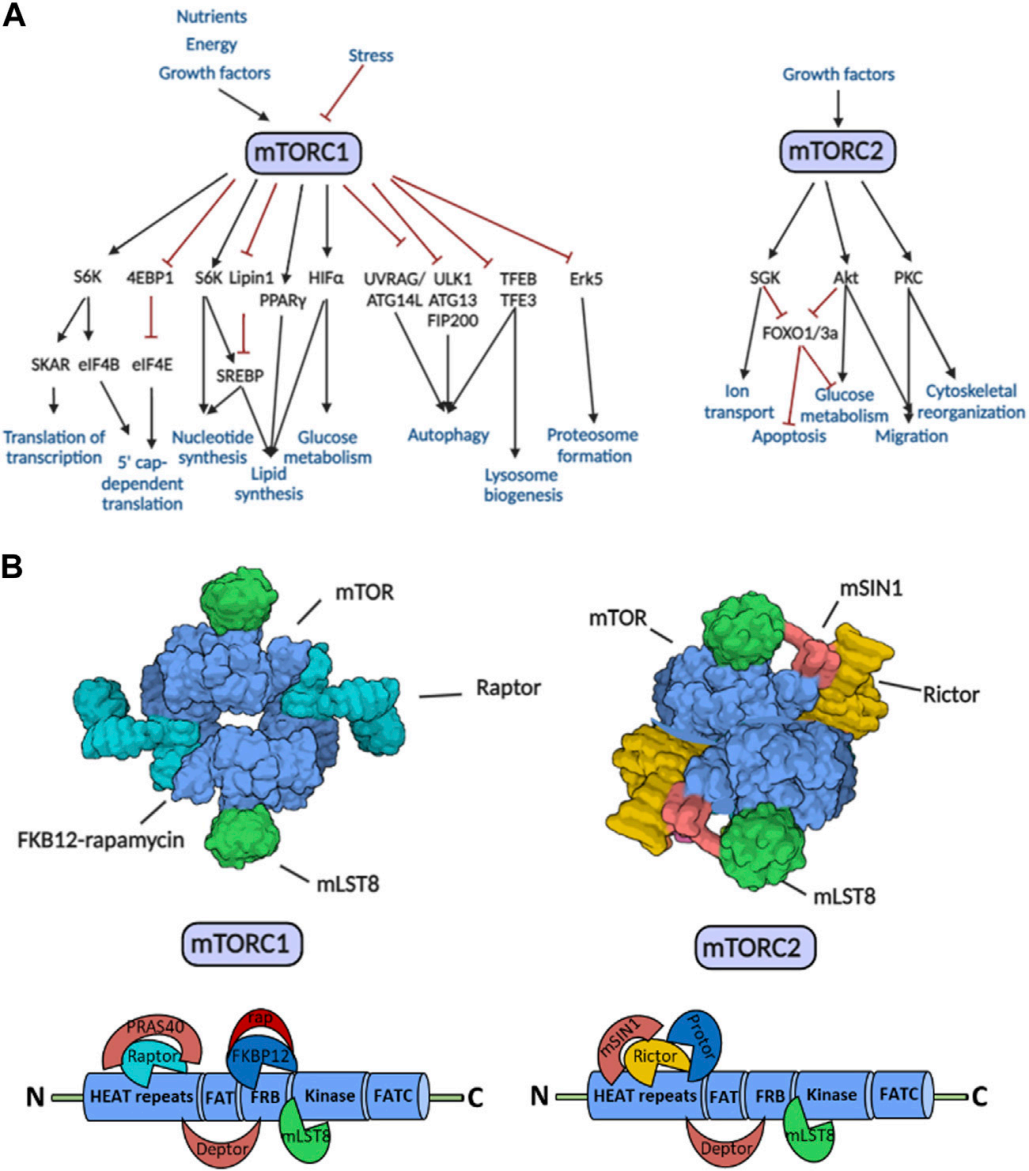
The regulatory roles, target proteins, and structure of the mTORC1 and mTORC2. **(A)** Effector mechanisms and functions of the mTORC1 and mTORC2. The schematic diagram shows the activating (black lines with arrows) and inhibitory (red lines with inhibition signs) effects. **(B)** Structural elements mTORC1 and mTORC2. The X-ray crystallographic field structure of mTOR complexes and the binding sites of specific partner molecules on the mTOR kinase domain are shown in the figure. Abbreviations: Deptor, DEP domain containing mTOR-interacting protein; HEAT repeats, Huntingtin, elongation factor 3 (EF3), protein phosphatase 2A (PP2A), TOR1; FAT domain, FRAP, ATM, TRRAP; FRB domain, FKBP12-rapamycin binding; FATC domain, C-terminal FAT domain; mLST8, mammalian lethal with sec-13 protein 8; mSin1, mammalian stress-activated map kinase-interacting protein 1; PRAS40, proline-rich Akt substrate of 40 kDa; Protor, protein observed with Rictor; rap, rapamycin; Raptor, regulatory-associated protein of mTOR; Rictor, rapamycin-insensitive companion of mTOR; other explanations in text.

The mechanistic (formerly mammalian) target of rapamycin (mTOR) influences various cellular processes (proliferation, motility, migration, metabolism, protein synthesis, transcription, etc.,) by integrating signals from the tissue environment. mTOR promotes cell growth and survival, depending on the actual state of the cell. In addition, a decrease in mTOR activity in the absence of growth factors, nutrients, or other energy sources (e.g., oxygen) inhibits cell growth and triggers survival-promoting cellular processes such as autophagy. As a central signaling pathway hub, mTOR kinase plays an essential role in metabolic regulation to maintain the balance between anabolic and catabolic processes, including metabolic adaptation in stress responses and tumor cell survival (Figure 1A). Dysfunction (hyper- or underactivation) of the mTOR kinase may contribute to regulatory failures and subsequently to the development and progression of diseases (e.g., metabolic, neurodegenerative, and cardiovascular diseases, accelerated aging, tumors) [1, 2].

mTOR is a serine-threonine kinase that forms two complexes (mTOR complex 1—mTORC1; mTOR complex 2—mTORC2) organized from different, functionally distinct proteins (Figure 1B). The identical subunits in the two complexes are mTOR kinase, mLST8 (mammalian lethal with sec-13 protein 8), and Deptor (DEP domain containing mTOR-interacting protein). PRAS40 (proline-rich Akt substrate of 40 kDa) and the scaffolding protein Raptor (regulatory-associated protein of mTOR) are involved in the assembly of mTORC1. mSin1 (mammalian stress-activated map kinase-interacting protein 1), Protor 1/2 (protein observed with Rictor 1 and 2), and the scaffolding protein Rictor (rapamycin-insensitive companion of mTOR) instead of Raptor are in mTORC2 [3].

mTORC1 plays a role in several cellular processes (e.g., protein, lipid and nucleotide synthesis, ribosome biogenesis), whereas mTORC2 has a significant role in the modulation of cell survival, differentiation, growth, and migration and the maintenance of the actin cytoskeleton, mainly through the phosphorylation of Akt (Ser473), PKCα, and SGK1 [4].

mTOR complexes and their dysfunction—mainly hyperactivity—have been associated with the development and progression of malignancies. Hyperactivity of mTOR complexes can occur in several ways; including gene mutations affecting mTOR kinase, mutations in proteins that directly regulate the mTOR complex activity, or other changes in the signaling network (e.g., mutations in oncogenic or tumor suppressor genes).

**FIGURE 2:**
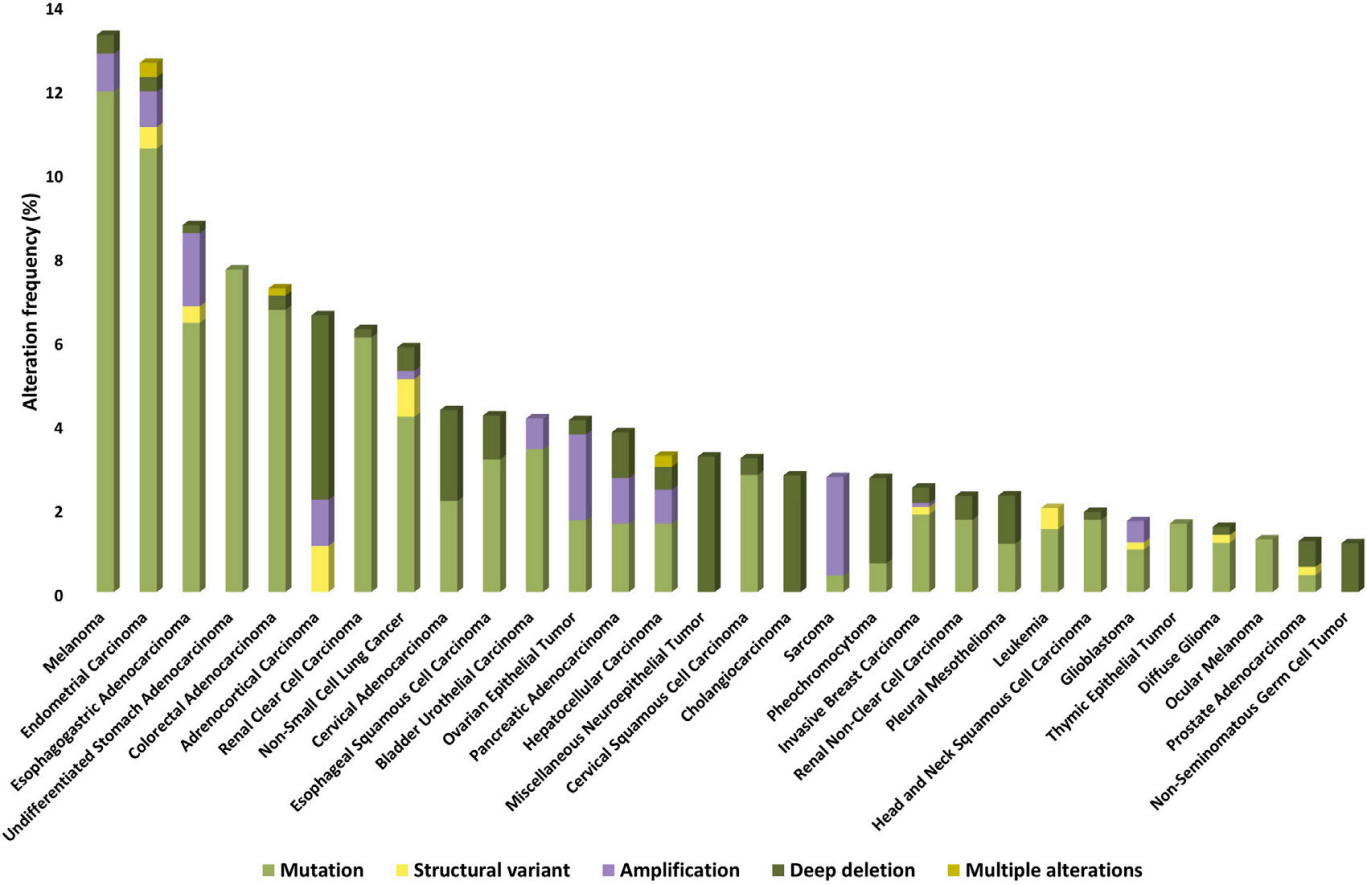
Prevalence of *MTOR* gene mutations, amplifications, deletions, and other structural gene defects in human tumors (based on TCGA PanCancer Atlas Studies databases).

Nearly 5% of solid tumors may carry an activating mutation of mTOR kinase, which may be more common (>5%) in melanoma, endometrial, gastrointestinal, kidney, breast, and lung cancers [5]. On the basis of publicly available databases, the frequencies of genetic alterations associated with/related to mTOR kinase in different malignancies are summarized in Figure 2.

**FIGURE 3:**
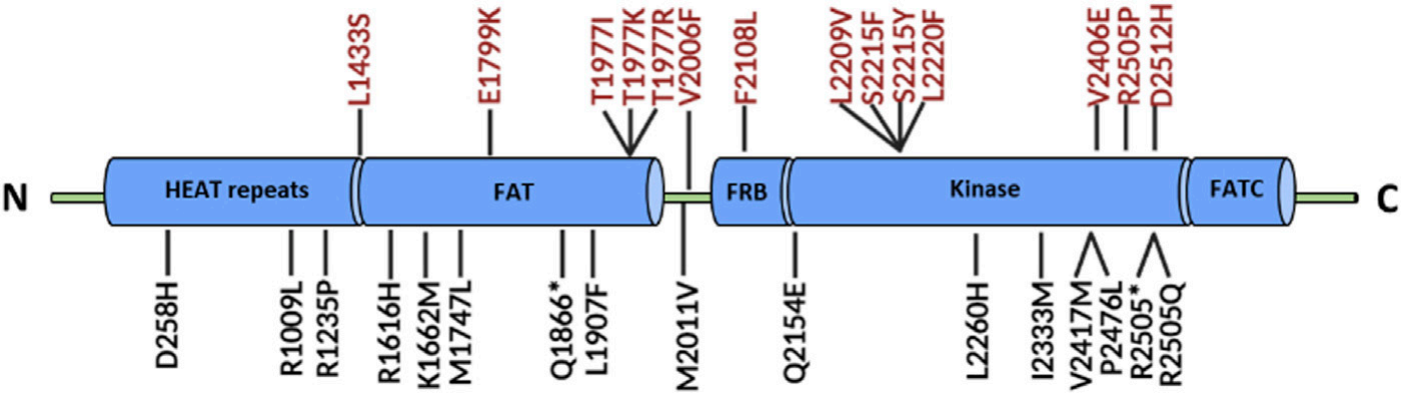
The structure of the mTOR kinase domain and some common mutations. The most common mutations responsible for changes in the structure of mTOR kinase are highlighted in red. Abbreviations: HEAT repeats, Huntingtin, elongation factor 3 (EF3), protein phosphatase 2A (PP2A), TOR1; FAT domain, FRAP, ATM, TRRAP; FRB domain, FKBP12-rapamycin binding; FATC domain, C-terminal FAT domain; other explanations in text.

The enzymatic activity and oncogenic role of mTOR kinase are generally increased by somatic mutations affecting six different kinase regions. The most common activating mutations are E1799K, T1977R, V2006F, S2215Y, and R2505P, which are responsible for amino acid substitutions (Figure 3). Activating mutations do not affect the assembly of the mTOR complex but can reduce the binding of Deptor—an inhibitor of mTOR kinase—and increase the phosphorylation of target molecules (e.g., P70S6K, 4EBP1) [6, 7]. Some mutations alter the structure of the complex, leading to resistance to allosteric mTOR inhibitors (e.g., rapamycin) by preventing drug binding. For instance, F2108L point mutation occurs in the FKBP-binding (FRB) domain of mTOR, where the FKB12-rapamycin binding site is located [8].

Most commonly, other mutations can cause pathway hyperactivity or loss of negative regulators in the signaling network (e.g., *PI3KCA*, *PTEN*, *TSC1/2*, *STK11*, and *AKT1*). *PI3KCA* mutations occur in more than 20% of breast and gynecological cancers and are also common in colorectal tumors. *PTEN* mutations are found in 10% of central nervous system and endometrial tumors, *TSC1* mutations in 5%–6% of urinary tract and endometrial tumors, and *TSC2* mutations in 4%–7% of gynecological, liver, and lung tumors. *STK11* mutations occur in 10%–15% of the cervical, small bowel, and lung tumors, while *AKT1* mutations are the least frequent of the signaling mutations (3%–5%). In many cases, mutations in receptor tyrosine kinases and growth factor receptors (EGFR and HER2) underline mTOR hyperactivity [9].

In addition to direct mutations in the mTOR kinase and the above-mentioned mutations affecting mTOR signaling, other gene mutations can occur in different subunits of the mTOR complex. One of the most common alterations is *RICTOR* amplification, which—in addition to increased expression of the Rictor protein—can increase the activity of the mTORC2 complex. In this context, it can affect/activate the target protein Akt kinase, which plays an essential role in the growth and survival of tumor cells (see below).

## *In situ* studies of mTOR activity

The discovery of mTOR complexes and the potential therapeutic relevance of the mTOR kinase inhibitor rapamycin focused attention on the need to characterize mTOR activity. First-generation allosteric mTOR inhibitors are effective in the case of mTORC1 hyperactivity, although these may be ineffective in specific known FRB domain mutations or the presence of high mTORC2 activity. Therefore, the development of a new generation of mTOR kinase inhibitors that inhibit both mTORC1 and mTORC2 and additional inhibitors such as dual inhibitors has been initiated. Based on many published data, it is necessary to characterize mTOR activity *in situ* before starting mTOR-targeted therapies, as the efficacy of drug treatment may also depend on the ratio of the complexes.

In our studies, we have developed marker panels that can be used in a wide range of tumor types to quantify and morphologically characterize not only mTORC1 but also mTORC2 complex activity in tumor cells or tissues. Characterization of mTOR activity in different cell lines and patient material can be performed by staining/measuring the active, phosphorylated form of mTOR kinase (p-mTOR) and its phosphorylated targets (e.g., p-4EBP1, p-p70S6K, p-S6) using immunohistochemistry (IHC)/immunocytochemistry and/or Western blot/Wes^TM^ Simple. One of the target proteins of the mTORC2 complex is the Akt kinase, where the amino acid serine 473 can only be phosphorylated by active mTORC2. On the basis of publicly available results, quantitative and *in situ* analysis of p-Akt (Ser473) is the most appropriate way to monitor the mTORC2 activity.

**FIGURE 4:**
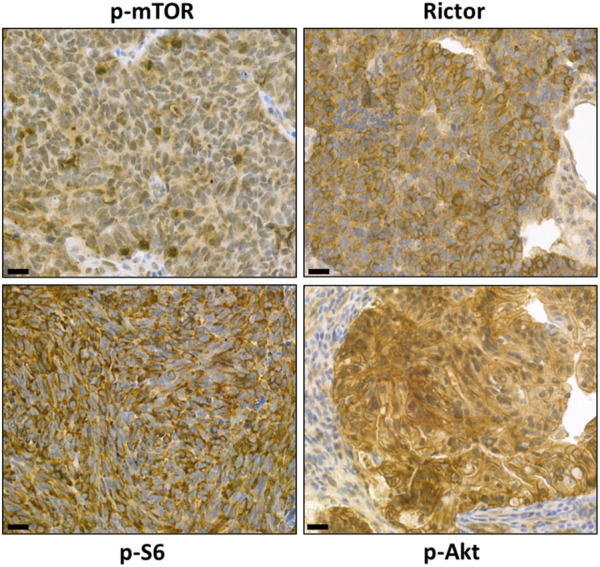
Representative images of characterizing mTORC1 and mTORC2 activity with our marker panel using immunohistochemistry. Detection of high levels of p-mTOR, p-S6, and Rictor *in situ* in brain metastases of small cell lung carcinoma and detection of p-Akt on paraffin sections of colorectal adenocarcinoma specimens. The magnifications are indicated, with 50 μm for IHC.

A quantitative comparison of the two mTOR complexes can also be determined from the ratio of specific scaffold proteins (Raptor for mTORC1 and Rictor for mTORC2). The optimal *in situ* marker panel is either a combination of p-mTOR, Raptor, and Rictor or a combination of mTORC1 target proteins (e.g., p-S6, p-70S6K, p-4EBP1) and a specific mTORC2 target protein [p-Akt (Ser473)] (Figure 4).

## mTOR and mTORC1 hyperactivity in malignant tumors

**FIGURE 5:**
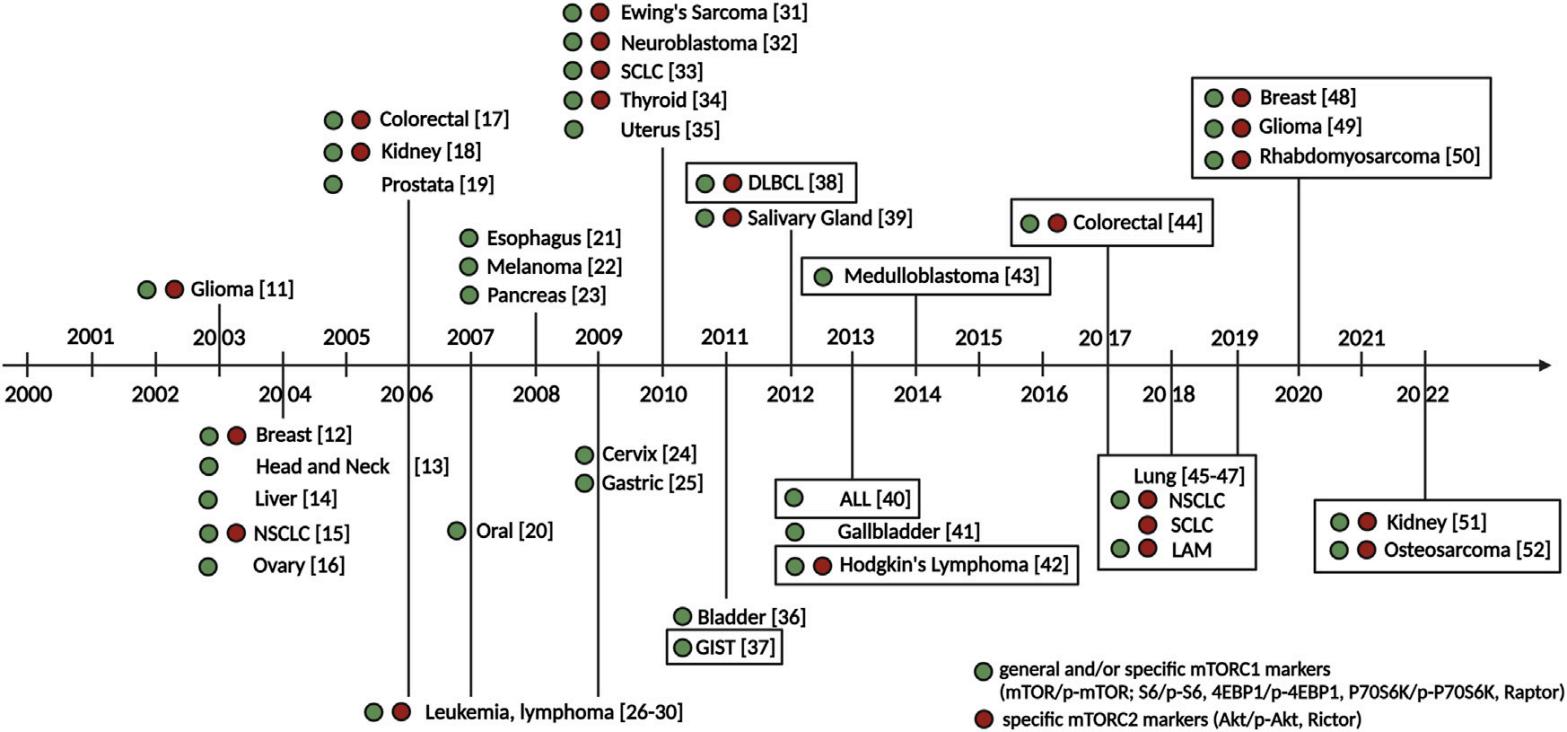
Milestones in the early characterization of mTOR hyperactivity in tumors. The first immunohistochemical characterizations of mTOR activity in the most common and later less common tumor types in the early 2000 s and our domestic characterizations (our work highlighted in a frame) are shown in the figure. Green and red circles indicate histopathological studies that detected general and/or specific mTORC1 complex markers (mTOR/p-mTOR; S6/p-S6, 4EBP1/p-4EBP1, P70S6K/p-P70S6K, Raptor) and specific mTORC2 complex markers (Akt/p-Akt, Rictor). Numbers indicate references. Abbreviations: ALL, acute lymphoblastic leukemia; DLBCL, diffuse large B-cell lymphoma; GIST, gastrointestinal stromal tumor; LAM, lymphangioleiomyomatosis; NSCLC, non-small cell lung cancer; SCLC, small cell lung cancer [11–52].

The first clinical anti-tumor observation of the mTOR inhibitor, the immunosuppressive drug rapamycin, was detected in the treatment of post-transplant kidney cancer [10]. Following the discovery of mTOR complexes, the mTOR hyperactivity and/or sensitivity of tumor cells against mTOR inhibitors, quantitative changes in the mTOR signaling pathway elements, target proteins, and their active forms have been described and partially characterized in various tumor types by the end of the last century and early 2000 s (detailed in Figure 5).

Our studies have contributed to the characterization of mTORC1 activity in hematological malignancies and even to the investigation of both mTOR complexes in leukemias and lymphomas. Summarizing these studies, acute lymphoblastic leukemia (ALL) cells and several lymphoma model cell lines (Hodgkin’s lymphoma, diffuse large B-cell lymphoma (DLBCL), anaplastic large cell lymphoma, mantle cell lymphoma, lymphoplasmacytic lymphoma, Burkitt’s lymphoma) show high mTORC1 activity. In addition to the generally high mTOR activity, the poorest survival outcomes were observed with the co-occurrence of high mTOR activity and increased Rictor expression. These findings highlight the unfavorable prognostic role of increased mTORC2 activity in the majority of lymphoid malignancies studied [38, 40, 42].

In addition to *in vitro* lymphoma models, mTOR hyperactivity has been characterized in the most common solid tumors and several human malignant tissues. In gastrointestinal tumors (colon tumors, gastrointestinal stromal tumors—GIST), we first characterized the activity of mTOR complexes, based on the amount of mTORC1 and mTORC2 scaffold proteins, and classified Hungarian colon tumor cases into three main groups (low Raptor and high Rictor expression—high mTORC2 activity; high Raptor and low Rictor expression—high mTORC1 activity; similar levels of Raptor and Rictor expression—balanced mTORC1 and mTORC2 activity). We compared our results with the clinical characteristics of the patients and found the best treatment and survival outcomes in cases with low mTORC1 activity, whereas the worst survival outcomes were observed in cases with high mTOR activity and high Rictor expression. Our results also showed that Rictor expression was associated with poor prognosis independently of mTOR activity [37, 44]. In our human breast cancer studies over the past few years, we have detected mTORC1 hyperactivity in 50% of our cases, supporting the use of previously introduced mTOR inhibitor therapy. In addition, we observed high Rictor expression—the increased presence of the mTORC2 complex—in more than 40% of the cases studied, which may explain the ineffectiveness of conventional rapalogue (rapamycin and its derivatives) treatments [48]. We also characterized mTOR activity in renal tumors. In our comparative studies, we could detect higher expression of p-mTOR and p-S6 markers in clear cell renal cell carcinoma compared to papillary renal cell carcinoma and normal tubular epithelial cells. Moreover, papillary renal cell carcinoma was characterized by high Rictor expression and potential mTORC2 activity, also highlighting the role of mTORC2 activity in this tumor type [51]. There has been an emerging need to characterize mTOR activity in rare solid tumors. Our studies also aimed to investigate the mTOR activity in rare tumor types, such as central nervous system tumors, childhood rhabdomyosarcoma, osteosarcoma, medulloblastoma, and fibrosarcoma [43, 49, 50, 52, 53].

## Overexpression of the Rictor protein and the associated increase in the amount and activity of mTORC2

Rictor is a characteristic actin coordinating scaffold protein of the mTORC2 complex, and its primary function is to ensure the formation and structure of the mTORC2 complex. Rictor contributes to the formation of a spatially structured mTORC2 complex in which the FKBP-rapamycin binding region of the mTOR kinase is located inside the molecule, blocking access to the best-known and most widely used inhibitor of rapamycin and its first-generation derivatives (rapalogs) [1]. Consequently, these inhibitors do not directly affect the mTORC2 target proteins (e.g., Akt). Increased Rictor expression is generally associated with transcriptional and translational changes. One of the best-known oncogenic mutations affecting the mTOR kinase pathway is the amplification of the *RICTOR* gene. *RICTOR* amplification can lead to increased expression of the Rictor protein and, in most cases, an increase in mTORC2 complex activity and a shift in the ratio of mTORC1 to mTORC2 complexes. *RICTOR* amplification has been described to play an essential role in the progression of certain tumors and metastasis via the regulation of signaling pathways (e.g., MAPK/ERK, Wnt/β-catenin pathways) [54].

**FIGURE 6:**
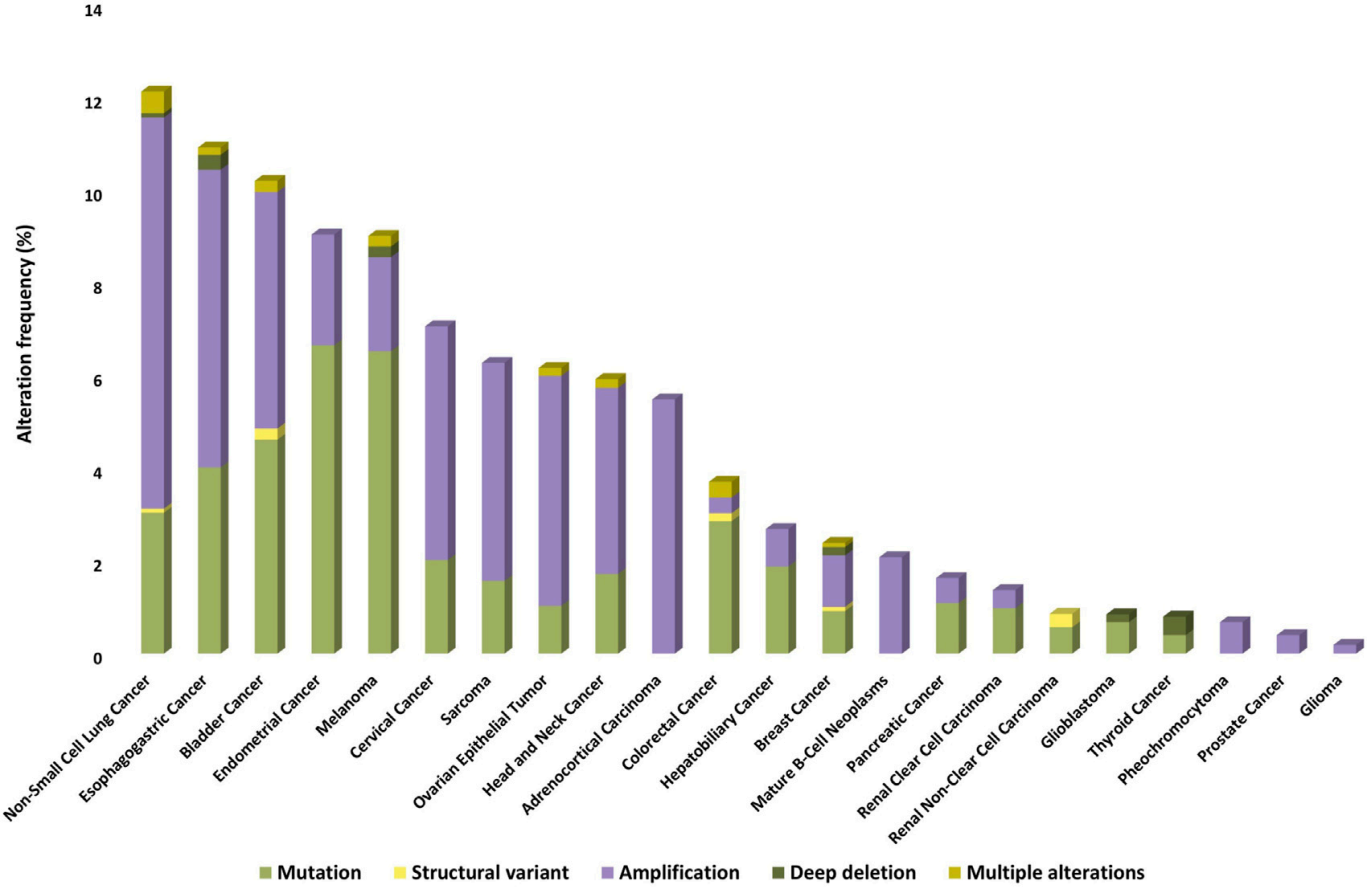
Prevalence of *RICTOR* gene mutations, amplifications, deletions, and other structural gene defects in human tumors (based on TCGA PanCancer Atlas Studies databases).

Among the genetic defects of *RICTOR*, the most frequently observed change is its amplification, but other alterations have also been described [55]. The genetic alteration frequency of *RICTOR* in different malignancies is summarized in Figure 6 (based on public databases).

Additionally, higher Rictor expression has been described in several tumor types, including lung [56], esophageal [57], breast [58], pancreatic [59], liver [60], and melanoma [61], where high Rictor expression was associated with poorer prognosis and shorter survival [55]. Table 1 summarizes mTORC2 hyperactivity and/or *RICTOR* amplification in different tumor types.

**TABLE 1:** Prevalence, prognostic, and therapeutic implications of Rictor expression and/or *RICTOR* amplification in various tumor types.

Tumor types		Data on Rictor expression and/or *RICTOR* amplification	Prognostic and therapeutic implications	Relevant publications
Breast Tumors	HER2+, Luminal A/B, and Triple-Negative Breast Cancer	37%–50% expression (IHC)	Increased mTORC2 activity is associated with an increased risk of metastasis and poorer prognosis	[48, 62–64]
	Metastatic Breast Cancer (lymph node)	—		[65]
Central Nervous System Tumors	Glioma, Glioblastoma	—	mTORC2 can be a potential therapeutic target.	[49, 66–69]
Digestive System Tumors	Colorectal Cancer	58% expression (IHC)	Increased Rictor expression is associated with therapy resistance, poorer prognosis, and shorter overall survival. Treatment with mTOR inhibitors may be beneficial in combination with other therapies (e.g., EGFR inhibitors, platinum-based chemotherapy)	[44, 70–73]
	Esophageal Squamous Cell Carcinoma	70% expression (IHC)		[57]
	Gastric Cancer	46%–78% expression (IHC), 38% amplification (FISH)		[73–75]
	Hepatocellular Carcinoma, Cholangiocarcinoma	40%–43% expression (IHC)		[73, 76, 77]
	Pancreatic Cancer	—		[59, 78]
Female Genital Tumors	Endometrial Cancer	44% expression (IHC)	Increased Rictor expression correlates with stage, metastasis, and poorer prognosis	[79]
Head and Neck Tumors	—	70% expression (IHC)	—	[80–82]
	HPV-associated Oral Squamous Cell Carcinoma	—	Increased Rictor expression is associated with a poorer prognosis	[83]
Other tumors	Pheochromocytoma	80% expression (IHC)	—	[84]
Skin Tumors	Melanoma	—	The presence of liver metastasis correlates with increased Rictor expression, and inhibition of mTORC2 may reduce metastasis formation	[61, 85]
Soft Tissue and Bone Tumors	—	28% expression (IHC), 20% expression (Western blot)	High mTORC2 activity is associated with a poorer prognosis	[73, 86]
	Myxofibrosarcoma	—		[87]
	Osteosarcoma	25% expression (IHC)		[52]
	Rhabdomyosarcoma	82% expression (IHC)		[50]
Thoracic Tumors	Lymphangioleiomyomatosis	55% expression (IHC)	—	[47]
	Metastatic Lung Cancer	66% expression (IHC)	—	[45]
	Non-Small Cell Lung Cancer	37% expression (IHC)	—	[45, 73]
	Small Cell Lung Cancer	14% expression (IHC), 6%–15% amplification (sequencing)	—	[46, 88–90]
Tumors of Haematopoietic and Lymphoid Tissues	Leukemia and Lymphoma (Acute Myeloid Leukemia, Acute Lymphoid Leukemia, Chronic Lymphocytic Leukemia, Chronic Myeloid Leukemia, Diffuse Large B-cell Lymphoma)	43%–63% expression (IHC)	High mTORC2 activity is associated with a poorer prognosis; inhibition of mTORC2 may be effective	[38, 42, 91–94]
Urinary and Male Genital Tumors	Bladder Cancer	—	Increased mTORC2 activity may affect the invasiveness of bladder cancer cells	[95]
	Kidney Cancer	47% expression (IHC)	Resistance to rapalogs is associated with increased mTORC2 activity	[51, 73, 96]

## Methods to study *RICTOR* amplification, Rictor expression, and mTORC2 activity

**FIGURE 7:**
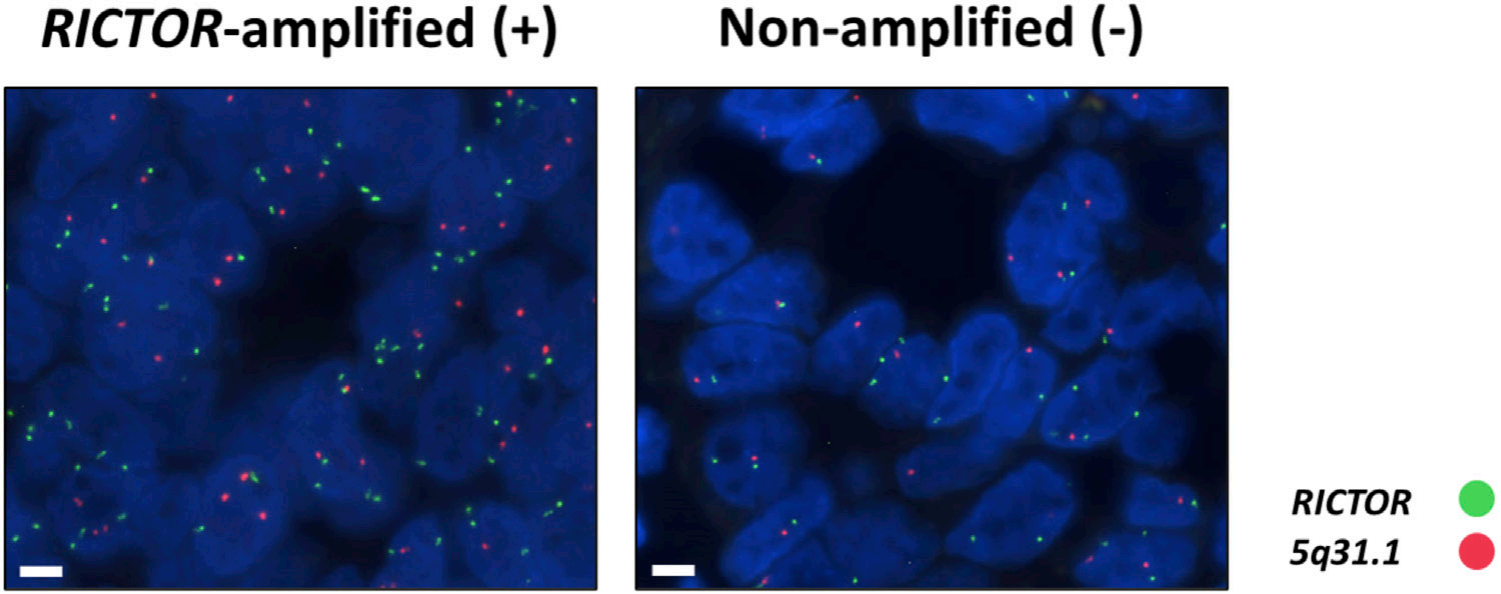
Representative images of the *RICTOR* FISH assay in *RICTOR*-amplified (+) and non-amplified (−) human tumor cases (*RICTOR* FISH was performed on the patient population of reference #97). The *RICTOR* (green) was used alongside the assay designed for the long arm (*5q31.1*) of chromosome 5 (orange) as a control (ZytoLight SPEC *RICTOR/5q31.1* Dual Color Probe—ZytoVision GmbH; Bremerhaven, Germany). The magnifications are indicated, with 5 μm for FISH.

*RICTOR* amplification may indicate high mTORC2 activity and may be a predictive marker of effective inhibition of the PI3K/mTOR/Akt axis. Fluorescence *in situ* hybridization (FISH) is considered the gold standard validation diagnostic method for identifying *RICTOR* amplification (Figure 7). In addition, potential changes in copy number can be detected by sequencing (e.g., next-generation sequencing) or Droplet Digital PCR (ddPCR). The Rictor protein expression can be detected by immunohistochemistry or immunocytochemistry. As mentioned above, the mTORC2 complex is exclusively responsible for the phosphorylation of serine 373 in the Akt protein. Increased p-Akt (Ser473) protein expression is a clear sign and an excellent marker of increased mTORC2 complex activity [46, 50]. It is important to note that tissue sample preparation and fixation require special attention when testing for p-Akt (Ser473) expression. Failures in the process and the timing of tissue sample fixation may reduce the detectability of phosphorylated proteins.

**FIGURE 8:**
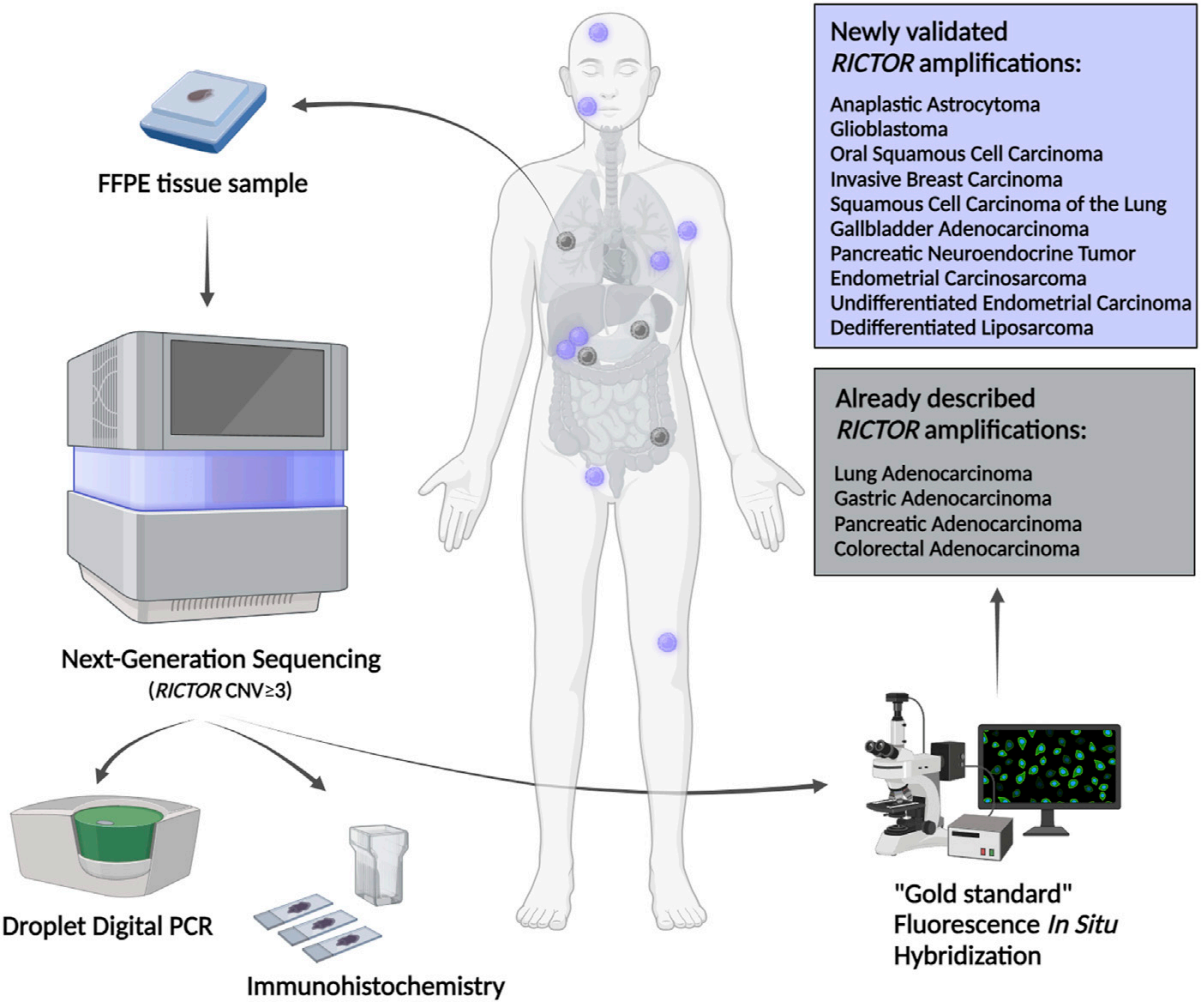
Newly described tumor types with FISH-validated *RICTOR* amplification. Abbreviations: FFPE, formalin-fixed, paraffin-embedded; CNV, copy number variation.

In our latest study, the diagnostic next-generation sequencing presumed *RICTOR* amplification was further analyzed in different human tumor tissues. *RICTOR* amplification was tested by ddPCR and validated using the “gold standard” FISH. In addition, Rictor and p-Akt (Ser473) protein expression was also examined by immunohistochemistry. The 10 novel and 4 previously described various tumor types with FISH-validated *RICTOR* amplification demonstrate the significance of *RICTOR* amplification in a wide range of malignancies (detailed in Figure 8). The newly described entities with *RICTOR* amplification may initiate further studies with larger cohorts to analyze the prevalence of *RICTOR* amplification also in rare diseases [97].

## mTOR hyperactivity and *RICTOR* amplification in lung tumors

Dysregulation of the PI3K/Akt/mTOR signaling pathway plays an important role in the development and progression of the majority of lung cancers [98]. mTOR activity can be affected by failures in other signaling pathways related to the mTOR pathway and the known alterations in mTOR pathway activity (see above). In this context, the combined inhibition of mTOR and other kinases may provide additional therapeutic options for treating lung tumors [56].

Among non-small cell lung cancers (NSCLC), 90% of adenocarcinomas, 40% of squamous cell carcinomas, and 60% of large cell carcinomas are characterized by increased activity of PI3K/Akt/mTOR axis [99]. Elevated levels of phosphorylated mTORC1 targets (e.g., p-4EBP1, p-S6) have been observed in adenocarcinoma and squamous cell carcinoma. In these studies, tumor invasiveness, metastasis, and poorer prognosis are associated with increased activity of mTOR [100]. Activating mutations in *PIK3CA* have been detected in 4%–7% of NSCLCs, amplification in more than 30% of squamous cell carcinomas, and about 1% of adenocarcinomas. *RICTOR* amplification has been described in 10% of NSCLCs, and mutations in the tumor suppressor genes *PTEN* and *STK11*, which are negative regulators of mTOR signaling, have also been observed in squamous cell and adenocarcinomas [101–103]. Moreover, mTOR activation correlates with mutations in both *KRAS* and epidermal growth factor receptor (EGFR) and may serve as a resistance mechanism to treatment with EGFR inhibitors [104].

In our studies, we examined the p-mTOR, p-S6, and Rictor proteins *in situ* in primary lung adenocarcinomas and brain metastases. Increased p-mTOR, p-S6, and Rictor protein expressions were observed in more than 30% of primary adenocarcinomas and around 70% of brain metastases. Additionally, all studied markers (p-mTOR, p-S6, and Rictor) showed a stronger positivity in the majority of the brain metastases studied. In some cases, high p-mTOR levels were associated with low p-S6 and high Rictor expression, which characterized about 20% of primary lung adenocarcinomas and more than 50% of brain metastases. Further associations were found between higher stage and Rictor expression in primary adenocarcinomas and high Rictor and low p-S6 expression in solitary brain metastases. Comparing primary tumor and brain metastases samples from the same patient, we observed increased mTORC1 activity in metastases; in 60% of the cases, p-mTOR and p-S6 expression was increased in brain metastases, whereas in 40% of cases, Rictor expression was increased in brain metastases. These data highlight the presence and prognostic role of mTORC2 activity in this tumor type [45].

Among lung tumors of neuroendocrine origin, small cell lung carcinoma (SCLC) is the most common, accounting for 15%–20% of all lung tumors [105]. In addition to mutations in the *TP53* and *RB1* genes and *MYC* amplification, genetic alterations affecting elements of the PI3K/Akt/mTOR pathway (e.g., *PIK3CA*, *PTEN*, *AKT2*, *AKT3*, *MTOR*, and *RICTOR*) are common in SCLC. *RICTOR* amplification is the most common targetable genetic alteration in SCLC (6%–14%) [106]. Activation of the mTOR pathway in SCLC has been detected by immunohistochemistry for p-mTOR and p-S6 proteins [33], although less data are available on the activity, background, and significance of mTORC2.

Our *RICTOR* amplification studies were initiated in collaboration with the Mayo Clinic, and within the framework of this collaboration, we analyzed 100 samples from 92 SCLC patients. The presence of *RICTOR* amplification was confirmed by FISH. We detected *RICTOR* amplification in 15% of cases, 3% of our cases were equivocal, and 82% were negative. In these samples, we characterized Rictor and p-Akt levels by immunohistochemistry. Of the cases, 14% showed high, 23% moderate, 25% low Rictor expression, and 38% were negative. The expression of p-Akt was high in 16%, moderate in 26%, and low in 35% of the cases, whereas in 23% of the tested samples, we could not detect the presence of the protein. Statistical analyses showed that Rictor immunohistochemistry had a sensitivity of 93% and a specificity of 73%; p-Akt immunohistochemistry had a sensitivity of 80% and a specificity of 65% compared to *RICTOR* FISH results. The presence or absence of *RICTOR* amplification was not associated with the overall survival data of the patients. However, higher *in situ* expression of Rictor and p-Akt proteins was significantly associated with shorter overall survival [46].

A rare lung disease, lymphangioleiomyomatosis (LAM), is caused by a loss-of-function mutation in the *TSC1/2* gene, resulting in lung tissue damage through proliferation, growth, and invasion of LAM cells [107, 108]. The therapeutic relevance of mTORC1 activity and the expression of downstream markers of the mTOR signaling pathway (e.g., p-p70S6K, p-S6, p-4EBP1) have been demonstrated by immunohistochemical studies [109, 110].

With the help of the Mayo Clinic, 11 cases of LAM were involved in our studies. Most of these (91%) showed high p-S6 protein expression, demonstrating increased mTORC1 activity and supporting mTOR inhibitor treatments. In parallel, high levels of Rictor expression were detected in addition to p-S6 in more than half of the cases (55%). This observation also highlights the role of the potential activity of the mTORC2 complex in this disease, which may explain the ineffectiveness of mTORC1 inhibitors in certain cases. This study was the first to examine both mTORC1 and mTORC2 activity in human LAM samples, and in addition to previously reported mTORC1 hyperactivity, we have also demonstrated increased mTORC2 complex presence and activity. Further studies demonstrating mTORC2 hyperactivity may suggest using other dual mTORC1 and mTORC2 inhibitors or dual mTOR inhibitors to slow the progression of this rare disease [47].

Our studies support the importance of characterizing mTOR activity in a wide range of tumors, which may indicate the clinical need for the appropriate use of mTOR inhibitors. In these cases, we recommend the immunohistochemical analysis of at least 3-4 *in situ* tissue markers [e.g., p-mTOR, p-S6, Rictor, and p-Akt (Ser473)] to provide a good representation of mTORC1 and mTORC2 activity in tumor tissue. It is important to note that first-generation mTORC1 inhibitors may be ineffective in the presence of high Rictor and p-Akt (Ser473) expression, as tumor cells with high mTORC2 activity may survive despite the administration of specific mTORC1 inhibitors [111].

## Targeted therapeutic options in the presence of mTOR hyperactivity and/or *RICTOR* amplification

Over the past decades, the efficacy of several PI3K/Akt/mTOR signaling inhibitors has been investigated. Despite numerous clinical trials with inhibitors of this signaling pathway, therapeutic responses are often lower than expected; moreover, only a few new drugs have been introduced to treat various tumors. One possible reason for this could be an inadequate patient selection method and, in this context, the lack of reliable predictive markers. Sensitivity to mTOR inhibitors may be predicted by specific genetic abnormalities (e.g., *PIK3CA* mutation/amplification, *PTEN* loss-of-function mutation, *AKT* mutation, *RICTOR* amplification) or overexpression of the signaling pathway elements and their active targets (e.g., Rictor, p-S6, p-Akt). These markers can be well assessed at the tissue level, and their presence or absence should be evaluated/considered when designing targeted therapies before specific inhibitors are used [112, 113].

The best-known mTOR inhibitor, rapamycin (or sirolimus), also the eponymous inhibitor of mTOR kinase, was discovered by George Nogrady, a Hungarian-born bacteriologist, in the Chilean territory of Easter Island (Rapa Nui) in 1964 [114]. The compound was isolated as a microbial antibiotic and introduced as an immunosuppressant in 1975 [115]. Following the discovery of the specific target protein of rapamycin, mTOR kinase, allosteric mTOR inhibitors, rapamycin and its derivatives (rapalogs—everolimus, temsirolimus, etc.,) have been used with varying results in several types of tumors (e.g., breast, kidney, endocrine, central nervous system). Rapalogs inhibit the activity of mTORC1 by binding to the FRB domain of mTOR kinase through the FKBP12 protein. Due to the structural differences between the two complexes (see above), no direct effect of rapamycin is observed for the mTORC2 complex [111]. Further studies are needed to determine the possible impact of rapamycin on the mTORC2 complex.

In addition to rapalogs, double mTORC1 and mTORC2 inhibitors (e.g., CC-115, sapanisertib, vistusertib), as well as dual mTOR kinase and another signaling kinase (e.g., PI3K) inhibitors (e.g., gedatolisib, paxalisib, samatolisib) are currently under development. Other specific Akt inhibitors are also being tested (e.g., afuresertib, capivasertib, ipatasertib, MK-2206, TAS-117, triciribine, uprosertib) and are currently in phase II and III trials. Third-generation mTOR inhibitors (e.g., RapaLink-1) are also being developed for the treatment of various advanced cancers (e.g., renal, breast, mantle cell, and other high-grade lymphomas) [116]. Table 2 summarizes PI3K/Akt/mTOR inhibitors currently in use and development.

**TABLE 2:** Classification, highest clinical phase, status, and application of PI3K/Akt/mTOR inhibitors in various tumor types based on the GlobalData database.

Target	Drug Name	Tumor types	Highest phase and ID number of the clinical trial	Status
PI3K inhibitors				
Non-subunit-specific PI3K inhibitor	AZD8186	Solid Tumor	Phase II—NCT04001569	Active
	buparlisib (NVP-BKM120)	Head and Neck Cancer	Phase III—NCT04338399	Active
	copanlisib	Lymphoma	—	Marketed
	duvelisib	Leukemia, Lymphoma	—	Marketed
	MEN1611 (CH5132799)	Colorectal Cancer	Phase II—NCT04495621	Active
	tenalisib (RP6530)	Breast Cancer	Phase II—NCT05021900	Active
	TQB-3525	Breast Cancer, Endometrial Cancer, Leukemia, Lymphoma, Ovarian Cancer	Phase II—NCT04324879, NCT04355520, NCT04398953, NCT04610970, NCT04615468, NCT04808570, NCT04836663	Active
PI3Kα inhibitor	alpelisib	Breast Cancer	—	Marketed
	CYH-33 (HHCYH-33)	Solid Tumor	Phase I—NCT04586335, NCT04856371	Active
	inavolisib (GDC-0077)	Breast Cancer	Phase III—NCT04191499	Active
	serabelisib (INK-1117)	Solid Tumor	Phase II—NCT04073680	Active
	taselisib	Lymphoma, Myeloma, Solid Tumor	Phase II—NCT02465060	Active
PI3Kβ inhibitor	GSK2636771	Lymphoma, Myeloma, Solid Tumor	Phase II—NCT02465060	Active
PI3Kγ inhibitor	eganelisib (IPI-549)	Breast Cancer, Kidney Cancer	Phase II—NCT03961698	Active
PI3Kδ inhibitor	amdizalisib (HMPL-689)	Lymphoma	Phase II—NCT04849351	Active
	idelalisib	Leukemia, Lymphoma	—	Marketed
	IOA-244	Lymphoma, Melanoma, Solid Tumor	Phase I—NCT04328844	Active
	linperlisib (YY-20394)	Lymphoma	Phase II—NCT04370405, NCT04379167, NCT04500561, NCT04705090, NCT04948788	Active
	parsaclisib (INCB50465)	Lymphoma, Myelofibrosis	Phase III—NCT04551053, NCT04551066, NCT04796922, NCT04849715	Active
	SHC014748 (SH-748)	Lymphoma	Phase II—NCT04431089, NCT04470141	Active
	umbralisib	Lymphoma	-	Marketed
	zandelisib (PWT-143)	Lymphoma	Phase III—NCT04745832	Active
Various targets	AMG319 (ACP-319), apitolisib, AZD-8835, bimiralisib (PQR309), dactolisib, dezapelisib (NCB-040093), nemiralisib (GSK2269557), pictilisib (GDC-0941), pilaralisib, SAR260301, seletalisib (UCB-5857), SF1126, sonolisib, voxtalisib, ZSTK-474etc.			Inactive/Discontinued
mTOR inhibitors				
Allosteric mTOR inhibitor	everolimus	Breast Cancer, Central Nervous System Tumor, Endocrine Tumor, Kidney Cancer	—	Marketed
	sirolimus	Lymphangioleiomyomatosis	—	Marketed
	temsirolimus	Kidney Cancer	—	Marketed
mTOR-kinase inhibitor (inhibits both mTORC1 and mTORC2)	CC-115	Central Nervous System Tumor	Phase II—NCT02977780	Active
	onatasertib (ATG-008)	Solid Tumor	Phase II—NCT03591965, NCT04337463, NCT04518137, NCT04998760	Active
	sapanisertib (MLN0128)	Lymphoma, Myeloma, Solid Tumor	Phase II—NCT02465060	Active
	vistusertib	Lung Cancer	Phase II—NCT02664935, NCT03334617	Active
Various targets	apitolisib, AZD8055, bimiralisib (PQR309), dactolisib, ridaforolimus (Deforolimus, MK-8669), SF1126, voxtalisibetc.			Inactive/Discontinued
Akt inhibitors				
Pan-Akt	afuresertib	Ovarian Cancer, Prostate Cancer	Phase II—NCT04060394, NCT04374630	Active
	capivasertib	Breast Cancer, Prostate Cancer	Phase III—NCT03997123, NCT04305496, NCT04493853, NCT04862663	Active
	ipatasertib	Breast Cancer, Prostate Cancer	Phase III—NCT03072238, NCT03337724, NCT04060862, NCT04177108, NCT04650581	Active
	MK-2206	Breast Cancer, Lung Cancer, Thymoma	Phase II—NCT01042379, NCT01306045	Active
	TAS-117	Solid Tumor	Phase II—NCT04770246	Active
	triciribine (PTX-200)	Leukemia	Phase II—NCT02930109	Active
	uprosertib	Myeloma, Solid Tumor	Phase II—NCT01902173, NCT01989598	Active
Various targets	COTI-2, LY-2503029, perifosineetc.			Inactive/Discontinued
Dual inhibitors				
PI3K, mTOR dual inhibitor	gedatolisib	Breast Cancer	Phase II—NCT03698383, NCT03911973	Active
	paxalisib	Central Nervous System Tumor	Phase III—NCT03970447	Active
	samotolisib	Lymphoma, Solid Tumor	Phase II—NCT03155620, NCT03213678	Active

Developing potent and highly selective small molecules specific to the mTORC2 complex while preserving mTORC1 activity remains challenging. Among the specific inhibitors of mTORC2, only a few inhibitors based primarily on siRNA technology (e.g., Rictor si-NP, JR-AB2-011) are under preclinical testing [64, 117].

Despite the association between PI3K/Akt/mTOR hyperactivation and adverse prognosis, the efficacy of mTOR inhibitors used as monotherapy is very low, as is the case for most targeted therapeutic agents. However, combining mTOR inhibitors with other targeted therapies or conventional chemotherapy/radiotherapy may help sensitize different agents and overcome resistance mechanisms to treatment. This type of combination therapy is also being developed for a wide range of malignancies (Table 3) [118]. However, the administration of these mTOR inhibitor combination therapies can also be limited by adverse effects and/or severe individual side effects that require very careful management by oncologists [119].

**TABLE 3:** Ongoing therapies in combination with mTOR inhibitors and their highest clinical trial phase in various tumor types based on the GlobalData database.

Tumor types	Therapy	Highest phase and ID number of the clinical trial
Breast Tumors		
Marketed: Metastatic, hormone receptor+/HER2− Breast Cancer (everolimus)		
HER2+ (hormone receptor−/HER2+) Breast Cancer	chemotherapy + inetetamab + sirolimus	Phase III—NCT04736589
Luminal A (hormone receptor+/HER2−) Breast Cancer	AZD2014/everolimus + fulvestrant	Phase II—NCT02216786
	everolimus + paclitaxel	Phase II—NCT04355858
Triple-Negative Breast Cancer	AZD2014 + AZD6244 (MEKI)	Phase I/II — NCT02583542
	bevacizumab + doxorubicin + everolimus	Phase II—NCT02456857
	AZD6244 + temsirolimus	Phase I—NCT00600496
Central Nervous System Tumors		
Marketed: Astrocytoma (everolimus)		
	perifosine + temsirolimus	Phase I—NCT02238496
	cyclophosphamide + dasatinib + temsirolimus	Phase I—NCT02389309
	temsirolimus + vorinostat	Phase I—NCT02420613
	irinotecan + nab-rapamycin + temozolomide	Phase I—NCT02975882
	everolimus + trametinib	Phase I—NCT04485559
	celecoxib + cyclophosphamide + etoposide + sirolimus	Phase II—NCT02574728
	nab-rapamycin + standard therapy	Phase II—NCT03463265
Digestive System Tumors		
Colorectal Cancer	AZD6244 + temsirolimus	Phase I—NCT00600496
	bevacizumab + FOLFOX6 + nab-rapamycin	Phase I/II—NCT03439462
Hepatocellular Carcinoma	everolimus + lenvatinib + trametinib	Phase II—NCT04803318
Pancreatic Cancer	gedatolisib + palbociclib	Phase I — NCT03065062
Head and Neck Tumors		
	gedatolisib + palbociclib	Phase I—NCT03065062
Neuroendocrine Tumors		
Marketed: Neuroendocrine Tumors of the Lungs, Pancreas, or Intestines (everolimus)		
Neurofibromatosis	PLX3397 (MTKI) + sirolimus	Phase I/II—NCT02584647
	selumetinib (MEKI) + sirolimus	Phase II—NCT03433183
	bevacizumab + everolimus + octreotide acetate	Phase II—NCT01229943
	everolimus + lenvatinib	Phase II—NCT03950609
Other Tumors		
Advanced Tumor	sirolimus/everolimus/temsirolimus + vorinostat	Phase I—NCT01087554
	bevacizumab + carboplatin/sorafenib/paclitaxel + temsirolimus	Phase I—NCT01187199
	everolimus + vandetanib	Phase I—NCT01582191
	ceritinib + everolimus	Phase I—NCT02321501
	cemiplimab + sirolimus/everolimus + prednisone	Phase I—NCT04339062
Hepatoblastoma	chemotherapy + temsirolimus	Phase III—NCT00980460
Neuroblastoma	irinotecan + temozolomide + temsirolimus	Phase II—NCT01767194
Solid Tumor	ixabepilone + temsirolimus	Phase I—NCT01375829
	cyclophosphamide + dasatinib + temsirolimus	Phase I—NCT02389309
	bevacizumab + cyclophosphamide + temsirolimus + valproát	Phase I—NCT02446431
	irinotecan + nab-rapamycin + temozolomide	Phase I—NCT02975882
	gedatolisib + palbociclib	Phase I—NCT03065062
	epacadostat + sirolimus	Phase I—NCT03217669
	celecoxib + cyclophosphamide + etoposide + sirolimus	Phase II—NCT02574728
	everolimus + lenvatinib + trametinib	Phase II—NCT04803318
Vascular Tumor	prednisolone + sirolimus	Phase II—NCT03188068
Skin Tumors		
Melanoma	AZD6244 + temsirolimus	Phase I—NCT00600496
Soft Tissue and Bone Tumors		
	chemotherapy + everolimus + temsirolimus	Phase I—NCT04199026
	nab-rapamycin + pazopanib hydrochloride	Phase I/II—NCT03660930
	everolimus + ribociclib	Phase II—NCT03114527
	chemotherapy + temsirolimus	Phase III—NCT02567435
Thoracic Tumors		
Marketed: Sporadic Lymphangioleiomyomatosis (sirolimus)		
Non-Small Cell Lung Cancer	gedatolisib + palbociclib	Phase I—NCT03065062
	epacadostat + sirolimus	Phase I—NCT03217669
	durvalumab + sirolimus	Phase I—NCT04348292
	AZD2014 + AZD6244 (MEKI)	Phase I/II—NCT02583542
Non-Small Cell Lung Cancer + Small Cell Lung Cancer	AZD6244 + temsirolimus	Phase I—NCT00600496
	auranofin + sirolimus	Phase I/II—NCT01737502
Tumors of Haematopoietic and Lymphoid Tissues		
Marketed: Mantle Cell Lymphoma (temsirolimus)		
Hodgkin’s Lymphoma	everolimus + itacitinib	Phase I/II—NCT03697408
Leukemia	decitabine + sirolimus	Phase I/II—NCT02109744
	azacitidine + sirolimus	Phase II—NCT01869114
	clofarabine + melphalan + sirolimus + tacrolimus	Phase II—NCT01885689
Urinary and Male Genital Tumors		
Marketed: Metastatic Kidney Cancer (everolimus, temsirolimus)		
Kidney Cancer	AZD6244 + temsirolimus	Phase I—NCT00600496
	DFF332 (HIF2αI) + everolimus	Phase I—NCT04895748
	sunitinib + temsirolimus	Phase II—NCT01517243
	everolimus + lenvatinib	Phase I—NCT03324373
		Phase II—NCT05012371
	anastrozol + AZD2014	Phase I/II—NCT02730923
	carboplatin + paclitaxel + temsirolimus	Phase II—NCT00977574
	everolimus + levonorgestrel	Phase II—NCT02397083
	everolimus + letrozole + ribociclib	Phase II—NCT03008408
	auranofin + sirolimus	Phase II—NCT03456700
	ATG008/ATG010	Phase II—NCT04998760

## Future perspectives

Advances in personalized therapies and the increasing availability of molecular genetic data on malignancies, including our findings of mTORC1 and mTORC2 activity and *RICTOR* amplification in various tumors, highlight the importance of validated targets in common and rare malignancies. The efficacy of targeted therapies often fails to deliver the expected results due to inadequate patient selection. In a biomarker-driven umbrella study, patients were selected based on *RICTOR* amplification, demonstrating a promising strategy for personalized therapies by selecting patients most likely to respond to targeted treatments. In this clinical trial, two of the four SCLC patients treated with a vistusertib (dual mTORC1 and mTORC2 inhibitor) showed an increase in survival of almost 1 year [120].

In our work, we have developed a marker panel to determine tissue characteristics of mTOR activity in biopsy specimens, patient materials, and cell lines. Our results and other molecular findings will hopefully support and optimize future therapeutic decisions.

mTOR hyperactivity can be a targeted genetic alteration in different tumor types. In our studies, we present our findings on the activity of both complexes and the characterization and prognostic role of mTORC2 complex hyperactivity. In the future, we intend to investigate further the significance of mTOR hyperactivity and *RICTOR* amplification in several malignancies. Expanding next-generation sequencing into routine diagnostics will facilitate more accurate oncogenic and tumor suppressor gene alterations mapping. Molecular pathology results and molecular genetics data will become widely available, which may help to identify mutations affecting mTOR activity. This will also help to determine the importance of specific mutations in rare tumor types.

In summary, several third-generation PI3K/Akt/mTOR pathway inhibitors are already developing, and some of these are in clinical trials. Only a few inhibitors have been approved for treating various cancers compared to the enormous amount of research and money spent on their development. To increase this number and to achieve clinical translation of more mTOR inhibitors or other targeted inhibitors into personalized therapies, it is essential to identify predictive markers that can help therapeutic decision-making. In conclusion, for biomarker-driven patient selection, it is crucial to develop agents that are as effective as possible while maintaining a good safety profile and use rational drug combinations that improve quality of life and may be effective in overcoming primary or acquired resistance.

## Footnotes

The figures in this review (Figures 1, 4, and 6) were edited using BioRender (https://biorender.com) under license from the Department of Pathology and Experimental Cancer Research, Semmelweis University.
